# Supplementary material for: Engineering Interfacial Environment of Epigallocatechin Gallate Coated Titanium for Next-Generation Bioactive Dental Implant Components
Source: Int J Mol Sci. 2023 Jan 31;24(3):2661. doi: 10.3390/ijms24032661 (PMC9917301; doi:10.3390/ijms24032661)
Supplement: Supplementary file 1 [file ijms-24-02661-s001.zip › ijms-2131266-supplementary.pdf]

Supplementary Material

High Performance Liquid Chromatography (HPLC) – Supplementary material

Release solution of implant uncoated, coated with PDA_EGCg and PDA_PEG_EGCg has been analyzed using HPLC methods to understand if antioxidant power was due to the release of EGCg polyphenolic molecule or directly by the polyphenol entrapped on the implant surface.

Briefly, uncoated and coated implant has been soaked in 2000 μL of water: ethanol 50:50 (v/v) solution for 2 h, after that the solution has been characterized using HPLC Shimadzu SLC 40 equipped with Shimadzu SPD-M40 diode array. Released solution were filtered 0.2 μm PTFE filter and analyzed using Kinetex Biphenyl (100x3.0 mm, 2.6 μm particle size) column from Phenomenex operated at 40 °C. The mobile phase consisted of 2% (v/v) acetic acid in water (mobile phase A, MPA) and 0.5% acetic acid in water and acetonitrile (50:50 v/v) (mobile phase B, MPB), and were used in the gradient method reported in Table 1, at a flow rate of 0.4 ml/min and total run time of 17 min.

Time (min)	MPA (%)	MPB (%)
0-3	100-70	0-30
3-8	70-40	30-60
8-11	40-100	60-100
11-13	0	100
13-14	0-70	100-30
14-16	70-100	30-0
16-17	100	0

Table S1. Details of the HPLC gradient method used for the PRPE analysis

The injection volume was 3 μL and the diode array operated at the wavelength range 200 to 600 nm. Possible polyphenols present in the solution were identified through comparison with reference compounds. The quantitation of each polyphenols was performed by using calibration curves of the corresponding reference compounds.

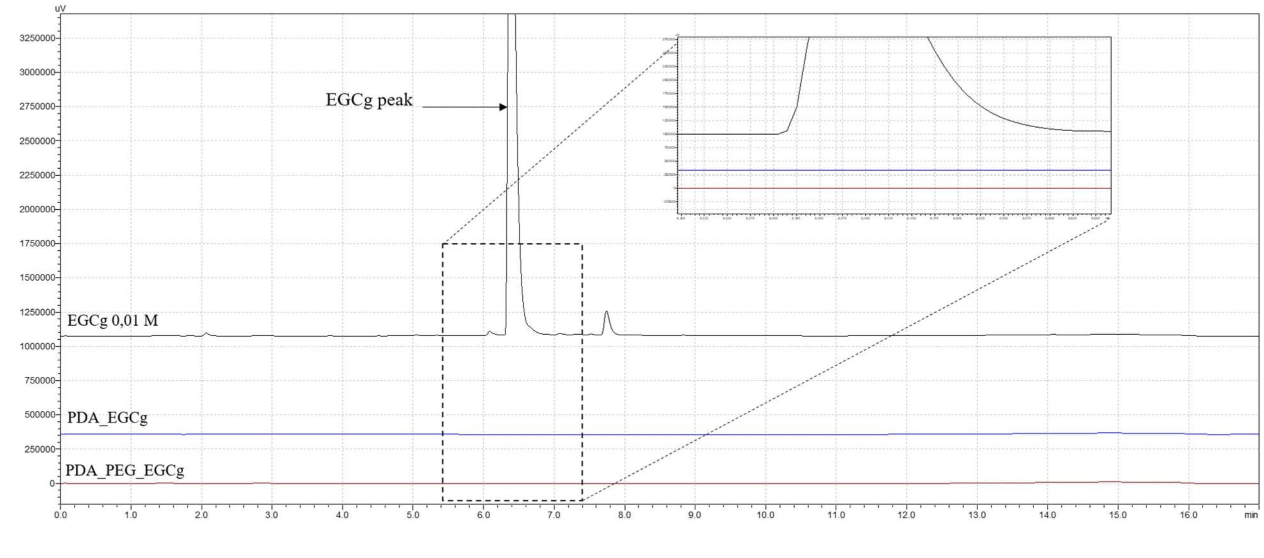

Figure S1. Chromatograms of EGCg 0.01 M solution, compared with the release solution after 2h from surface samples functionalized with PDA_EGCg and PDA_PEG_EGCg.

As is possible to note in Figure S1, the released solution from surface treated with PDS_EGCg and PDA_PEG_EGCg doesn't show any peak from EGCg molecules, compared with the chromatogram of EGCg 0.01 M solution. This means that the antioxidant power measured is due to the effect of the polyphenolic molecule entrapped on the surface and not due to the release of it.
